# N-*p*-*trans*-Coumaroyltyramine Improves Myocardial Ischemia–Reperfusion Injury: From Cellular Protection to Animal Model Validation and the Discovery of the Target Tcap

**DOI:** 10.3390/ijms27083523

**Published:** 2026-04-15

**Authors:** Xiangyun Chen, Yuxin Lu, Yunfang Kou, Mengyue Guo, Yaofeng Li

**Affiliations:** 1 School of Basic Medicine, Guizhou University of Traditional Chinese Medicine, Guiyang 550025, China; chenxiangyun017@gzy.edu.cn (X.C.); 18685817028@163.com (Y.L.); ivykou789@163.com (Y.K.); 2 School of Traditional Chinese Medicine, Guizhou University of Traditional Chinese Medicine, Guiyang 550025, China; 19912820157@163.com

**Keywords:** N-*p*-*trans*-Coumaroyltyramine, myocardial ischemia–reperfusion injury, myocardial protection, label-free quantitative proteomics, Titin-cap

## Abstract

Myocardial ischemia–reperfusion injury (MIRI) significantly limits the clinical benefits of reperfusion therapy, underscoring a pressing need for effective interventions. This study examines the cardioprotective effects and underlying mechanisms of the natural amide alkaloid N-*p*-*trans*-Coumaroyltyramine (p-CT). Using hypoxia/reoxygenation (H/R) models in neonatal rat cardiomyocytes and in vivo rat MIRI models, we assessed p-CT pretreatment on cell viability, cardiac function, serum injury markers (lactate dehydrogenase, creatine kinase-MB, cardiac troponin T, and myoglobin), myocardial histopathology, ultrastructural alterations, and infarct size. The systematic screening and validation of potential targets were conducted via label-free quantitative proteomics, molecular docking, and Western blot. The results demonstrated that p-CT pretreatment dose-dependently mitigated H/R-induced cellular injury, improved cardiac function in MIRI rats, reduced serum markers of myocardial damage, alleviated pathological and ultrastructural injury in myocardial tissue, and significantly diminished infarct size. Proteomic analysis revealed 19 differentially expressed proteins specifically reversed by p-CT, with Titin-cap (Tcap) exhibiting the most pronounced downregulation in the MIRI model—a change effectively restored by p-CT pretreatment. Molecular docking indicated strong binding affinity between p-CT and Tcap protein. In summary, p-CT represents a promising cardioprotective agent, likely exerting its effects by targeting Tcap protein and upregulating its expression, thereby helping preserve cardiomyocyte structural and functional integrity.

## 1. Introduction

Ischemic heart disease, particularly acute myocardial infarction (AMI), stands as a major global contributor to mortality and long-term disability [1,2]. For individuals experiencing an AMI, prompt restoration of coronary blood flow—achieved through percutaneous coronary intervention or thrombolytic therapy—is essential to salvage jeopardized myocardium and enhance clinical outcomes [3]. Nevertheless, the reperfusion process exhibits a dual-edged nature: while reestablishing circulation, it can also trigger a complex cascade of additional pathological damage, known as myocardial ischemia–reperfusion injury (MIRI) [4]. This injury may manifest as myocardial stunning, reperfusion-related arrhythmias, microvascular dysfunction, and cardiomyocyte death, substantially diminishing the therapeutic efficacy of reperfusion strategies and emerging as a critical constraint on long-term cardiac functional recovery [5]. Consequently, a deeper understanding of the pathophysiological mechanisms underlying MIRI, alongside the development of effective interventional approaches, carries considerable clinical relevance.

The pathophysiology of MIRI is characterized by a highly intricate cascade of reactions, where multiple factors and pathways interact in a complex network. Central to this process are oxidative stress, calcium overload, inflammatory responses, mitochondrial dysfunction, and the activation of diverse programmed cell death pathways [6,7,8,9]. The contractile function of cardiomyocytes relies on precisely organized myofibrillar structures, with actin and myosin filaments arranged in a regular pattern. In MIRI, calcium overload stimulates calcium-dependent proteases such as calpain, which degrade critical myofibrillar proteins including troponin, α-actinin, and connectin (titin) [10]. This proteolytic activity directly results in myofibrillar rupture and the disassembly of their ordered architecture, forming the structural basis for post-reperfusion myocardial stunning—manifested as persistent contractile dysfunction—and the progression toward heart failure [10]. Moreover, the activation of programmed cell death pathways, such as pyroptosis and necroptosis, further undermines cellular integrity. In pyroptosis, Gasdermin D forms membrane pores, while in necroptosis, mixed lineage kinase domain-like pseudokinase (MLKL) proteins mediate membrane rupture, both leading to cellular swelling, leakage of intracellular contents, and the physical disintegration of muscle fiber structures [8,11,12]. This breakdown releases additional damage-associated molecular patterns, amplifying inflammatory responses and creating a self-perpetuating vicious cycle [13]. Disorganized or ruptured muscle fibers are not only direct structural outcomes of intracellular events like protease activation and cell death execution, but also key markers in the transition from reversible contractile impairment to irreversible ventricular remodeling and heart failure. Thus, future therapeutic strategies should aim not only to target these core pathological processes to salvage cardiomyocyte viability, but also to focus on effectively safeguarding and restoring the contractile structural units of myocardial cells, which is essential for improving cardiac function following MIRI.

Natural products and their bioactive constituents, due to their structural diversity, multi-target effects, and favorable safety profiles, hold significant promise for the development of cardiovascular therapeutics [14,15]. Amides, a key class of natural alkaloids, are extensively distributed in plant families such as Piperaceae and Rutaceae. Research has validated their diverse pharmacological properties, including anti-inflammatory, anti-tumor, anti-parasitic, anti-gout, anti-diabetic, and cellular aging modulation [16,17,18,19,20]. N-*p*-*trans*-Coumaroyltyramine (p-CT) is a natural amide alkaloid structurally synthesized through an amide bond linkage between p-coumaric acid and tyramine [21]. Prior studies have demonstrated that p-CT exhibits multiple biological activities, such as α-glucosidase inhibition, anti-tumor effects, neuroprotection, immune modulation, and antioxidant properties [22,23,24,25]. Its mechanisms of action typically involve interactions with pivotal target proteins like AKT serine/threonine kinase 1 (AKT1), epidermal growth factor receptor (EGFR), cyclin D1 (CCND1), and phosphatidylinositol 3-kinase (PI3K), thereby regulating associated signaling pathways [23,24,26]. However, the potential cardioprotective effects of p-CT against MIRI, along with its specific pharmacodynamic characteristics and underlying molecular mechanisms, remain insufficiently explored.

To address this, the present study employs primary neonatal rat cardiomyocytes to establish a hypoxia/reoxygenation (H/R) model, combined with in vivo rat models of MIRI. Using label-free quantitative proteomics techniques, the research aims to evaluate the cardioprotective efficacy of p-CT and preliminarily investigate its mechanisms. The objective is to elucidate the pharmacological efficacy of p-CT in protecting against myocardial ischemia–reperfusion injury and to provide insights into its molecular basis, thereby laying a theoretical and practical foundation for the potential development of p-CT as a cardioprotective agent.

## 2. Results

### 2.1. Effects of p-CT on the Viability of Neonatal Rat Cardiomyocytes and Screening of Optimal Protective Concentration

**Figure 1:**
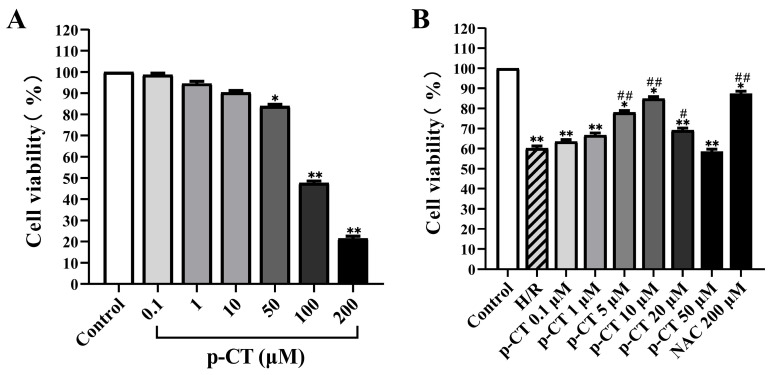
The effect of p-CT on the viability of normal and hypoxic–reoxygenated neonatal rat cardiomyocytes. Data are presented as mean ± SD (*n* = 6 per group). (**A**) The impact of different concentrations of p-CT on the viability of neonatal rat cardiomyocytes. (**B**) The effect of pretreatment with various concentrations of p-CT on the viability of hypoxic–reoxygenated neonatal rat cardiomyocytes. Compared to the control group, * *p* < 0.05, ** *p* < 0.01. Compared to the H/R group, # *p* < 0.05, ## *p* < 0.01.

To establish a safe concentration range of p-CT for further experimental use, this study first assessed its direct cytotoxic effects on neonatal rat cardiomyocytes via the cell counting kit-8 (CCK-8) assay. As shown in Figure 1A, p-CT did not markedly impair cell viability at concentrations ranging from 0.1 to 10 μM. Beyond this range, however, cell viability declined in a dose-dependent manner. Notably, in the 100 μM and 200 μM treatment groups, viability was significantly reduced compared to the control group (*p* < 0.01), demonstrating that elevated concentrations of p-CT induce clear cytotoxic responses.

Based on the toxicity screening results, this study utilized p-CT at concentrations of 50 μM and lower to evaluate its protective actions against hypoxia–reoxygenation (H/R) injury in neonatal rat cardiomyocytes. As depicted in Figure 1B, CCK-8 assay data revealed that H/R exposure markedly decreased cell viability relative to the normal control group (*p* < 0.01), indicating that H/R treatment successfully induced cardiomyocyte injury, with reduced cell viability serving as a key hallmark of this simulated ischemia–reperfusion model. Pretreatment with increasing concentrations of p-CT (0.1–10 μM) prior to H/R insult dose-dependently attenuated cellular damage and significantly restored cell viability. Notably, pretreatment with 5 μM and 10 μM p-CT exhibited pronounced cardioprotective efficacy (*p* < 0.01). Although the observed value remained statistically lower than that of the control group (*p* < 0.05), it was significantly improved compared to the H/R group (*p* < 0.01). In contrast, the protective effect diminished at 20 μM p-CT, and no significant benefit was observed with 50 μM pretreatment. For comparison, the antioxidant N-acetylcysteine (NAC, 200 μM), employed as a positive control, also significantly enhanced cell survival (*p* < 0.01). Collectively, these findings demonstrate that p-CT effectively protects neonatal rat cardiomyocytes from H/R-induced injury within the 5–10 μM concentration window, thereby providing a rationale for concentration selection in subsequent experiments.

### 2.2. Pretreatment with p-CT Attenuates H/R-Induced Release of Myocardial Injury Markers LDH and CK-MB in Neonatal Rat Cardiomyocytes

**Figure 2:**
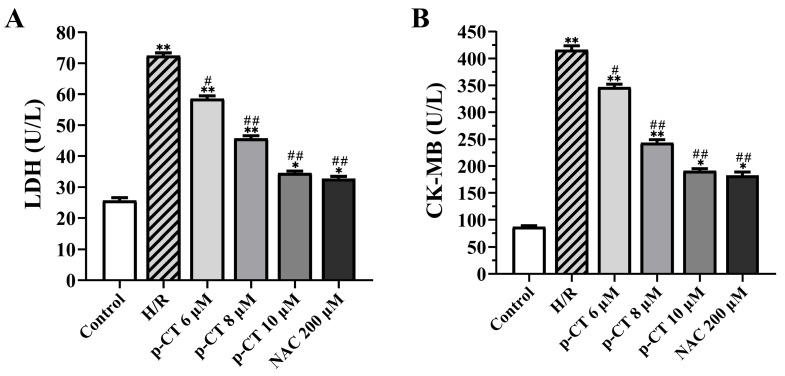
The effect of p-CT on the levels of lactate dehydrogenase (LDH) and creatine kinase isoenzyme MB (CK-MB) in the supernatant of H/R-injured neonatal rat cardiomyocytes. Data are presented as mean ± SD (*n* = 3 per group). (**A**) LDH activity in the supernatant of cell cultures from each group. (**B**) CK-MB activity in the supernatant of cell cultures from each group. Compared to the control group, * *p* < 0.05, ** *p* < 0.01. Compared to the H/R group, # *p* < 0.05, ## *p* < 0.01.

To evaluate the extent of cardiomyocyte injury, this study measured the activity of two critical injury biomarkers—lactate dehydrogenase (LDH) and the myocardial-specific creatine kinase isoenzyme MB (CK-MB)—in the culture supernatant of neonatal rat cardiomyocytes. As illustrated in Figure 2, compared with the Control group, both LDH activity (Figure 2A) and CK-MB activity (Figure 2B) were significantly elevated in the H/R group supernatant (*p* < 0.01), demonstrating that H/R treatment successfully compromised cardiomyocyte membrane integrity and provoked the substantial leakage of intracellular enzymes. In contrast, pretreatment with varying concentrations of p-CT (6, 8, 10 μM) markedly suppressed the H/R-induced release of LDH and CK-MB (*p* < 0.01 or *p* < 0.05), with the inhibitory effect exhibiting a clear dose-dependent pattern. Notably, the protective efficacy of 10 μM p-CT was comparable to that of 200 μM NAC, with no statistically significant difference observed between these two treatment groups (*p* > 0.05). These findings indicate that p-CT pretreatment effectively mitigates the H/R-triggered leakage of myocardial-specific enzymes, likely through the stabilization of the cardiomyocyte membrane structure, thereby conferring cytoprotection.

### 2.3. Pretreatment with p-CT Improves Cardiac Function in Rats with MIRI

**Figure 3:**
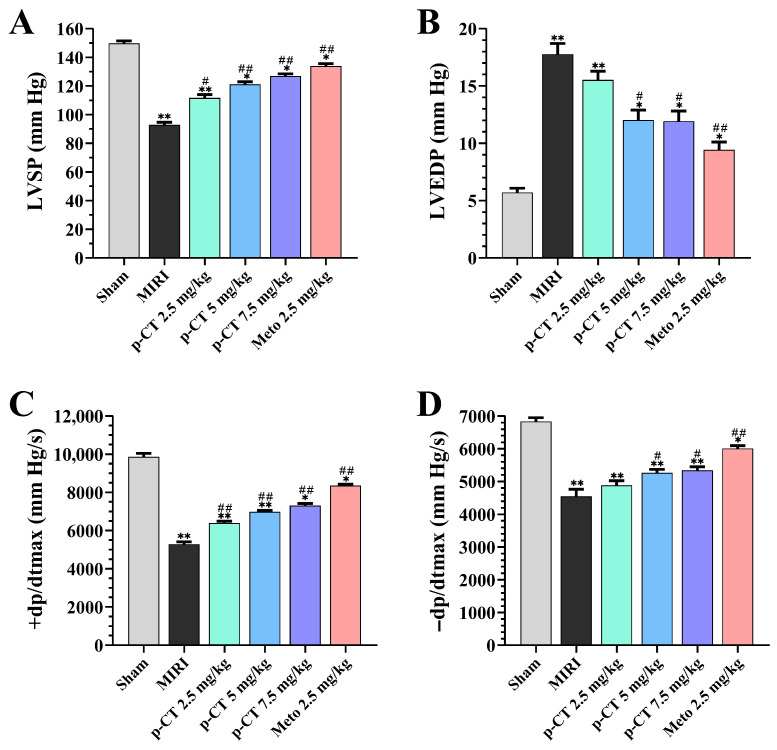
Effects of p-CT pretreatment on cardiac function following myocardial ischemia–reperfusion injury in rats. Data are presented as mean ± SD (*n* = 9). (**A**) Left ventricular systolic pressure (LVSP). One-way ANOVA followed by Tukey’s post hoc test: F(5, 48) = 285.7, *p* < 0.0001. * *p* < 0.05, ** *p* < 0.01 vs. sham group; # *p* < 0.05, ## *p* < 0.01 vs. MIRI group. (**B**) Left ventricular end-diastolic pressure (LVEDP). One-way ANOVA followed by Tukey’s post hoc test: F(5, 48) = 117.2, *p* < 0.0001. * *p* < 0.05, ** *p* < 0.01 vs. sham group; # *p* < 0.05, ## *p* < 0.01 vs. MIRI group. (**C**) Maximum rate of left ventricular pressure increase (+dp/dtmax). One-way ANOVA followed by Tukey’s post hoc test: F(5, 48) = 312.5, *p* < 0.0001. * *p* < 0.05, ** *p* < 0.01 vs. sham group; ## *p* < 0.01 vs. MIRI group. (**D**) Maximum rate of left ventricular pressure decrease (−dp/dtmax). One-way ANOVA followed by Tukey’s post hoc test: F(5, 48) = 185.4, *p* < 0.0001. * *p* < 0.05, ** *p* < 0.01 vs. sham group; # *p* < 0.05, ## *p* < 0.01 vs. MIRI group.

To assess the cardioprotective potential of p-CT at the whole-animal level, a rat model of MIRI was established, and key cardiac function parameters were evaluated. As illustrated in Figure 3, compared with the sham group, the MIRI model group displayed significant cardiac dysfunction, characterized by a marked reduction in left ventricular systolic pressure (LVSP; Figure 3A) and in the maximum rates of left ventricular pressure increase and decrease (±dp/dtmax; Figure 3C,D) (*p* < 0.01), alongside a significant elevation in left ventricular end-diastolic pressure (LVEDP; Figure 3B) (*p* < 0.01). These findings indicate that MIRI severely compromises both the systolic and diastolic performance of the rat heart. Intravenous pretreatment with varying doses of p-CT (2.5, 5, and 7.5 mg/kg) produced dose-dependent improvements in these functional indices. Relative to the MIRI group, medium and high doses of p-CT (5 and 7.5 mg/kg) significantly restored the depressed LVSP (*p* < 0.01; Figure 3A) and ±dp/dtmax (*p* < 0.01; Figure 3C,D), while also significantly lowering the pathologically raised LVEDP (*p* < 0.05; Figure 3B). Although the value remained statistically different from that in the sham group (*p* < 0.05), it was significantly improved compared to the MIRI group. Comparable cardioprotection was observed with pretreatment using the positive control drug Metoprolol (2.5 mg/kg). The protective effect of 7.5 mg/kg p-CT on the aforementioned indices was comparable to that of 2.5 mg/kg Metoprolol, with no statistically significant difference observed (*p* > 0.05). Together, these results demonstrate that intravenous p-CT pretreatment effectively enhances cardiac pumping function in MIRI rats, improving both myocardial contractility and relaxation capacity.

### 2.4. Impact of p-CT Pretreatment on Serum Myocardial Injury Marker Levels in a Rat Model of MIRI

**Figure 4:**
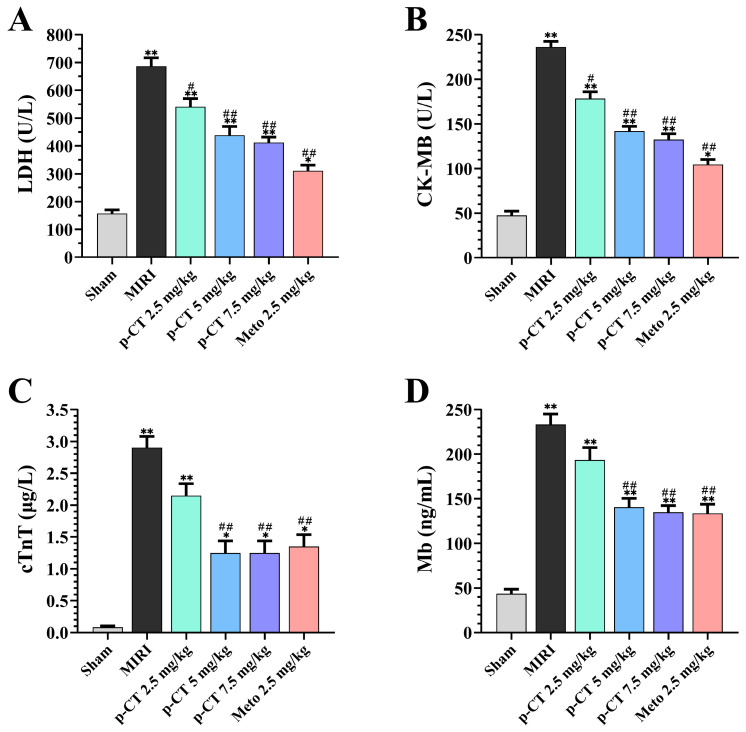
Effects of p-CT pretreatment on serum levels of myocardial injury markers following MIRI in rats. Data are presented as mean ± SD (*n* = 9). (**A**) Lactate dehydrogenase (LDH) activity. One-way ANOVA followed by Tukey’s post hoc test: F(5, 48) = 332.5, *p* < 0.0001. * *p* < 0.05, ** *p* < 0.01 vs. sham group; # *p* < 0.05, ## *p* < 0.01 vs. MIRI group. (**B**) Creatine kinase isoenzyme MB (CK-MB) activity. One-way ANOVA followed by Tukey’s post hoc test: F(5, 48) = 285.7, *p* < 0.0001. * *p* < 0.05, ** *p* < 0.01 vs. sham group; # *p* < 0.05, ## *p* < 0.01 vs. MIRI group. (**C**) Cardiac troponin T (cTnT) concentration. One-way ANOVA followed by Tukey’s post hoc test: F(5, 48) = 415.8, *p* < 0.0001. * *p* < 0.05, ** *p* < 0.01 vs. sham group; ## *p* < 0.01 vs. MIRI group. (**D**) Myoglobin (Mb) concentration. One-way ANOVA followed by Tukey’s post hoc test: F(5, 48) = 198.6, *p* < 0.0001. ** *p* < 0.01 vs. sham group; ## *p* < 0.01 vs. MIRI group.

As illustrated in Figure 4, relative to the sham-operated group, the MIRI model group exhibited marked elevations in serum levels of lactate dehydrogenase (LDH, Figure 4A), creatine kinase isoenzyme MB (CK-MB, Figure 4B), cardiac troponin T (cTnT, Figure 4C), and myoglobin (Mb, Figure 4D), with all increases reaching statistical significance (*p* < 0.01). These findings confirm that the ischemia–reperfusion procedure induced substantial cardiomyocyte necrosis and compromised sarcolemmal integrity. In comparison to the MIRI model group, intravenous pretreatment with varying doses (2.5, 5, and 7.5 mg/kg) of p-CT prior to the ischemic insult dose-dependently suppressed the leakage of these injury markers into the serum. Specifically, medium and high doses (5 and 7.5 mg/kg) of p-CT pretreatment significantly attenuated serum LDH activity (*p* < 0.01, Figure 4A) and CK-MB activity (*p* < 0.01, Figure 4B). Furthermore, serum concentrations of the highly specific and sensitive myocardial injury indicators cTnT (Figure 4C) and Mb (Figure 4D) were also significantly and dose-dependently reduced in the medium- and high-dose p-CT pretreatment groups (*p* < 0.01). The positive control drug Metoprolol (2.5 mg/kg) similarly demonstrated significant cardioprotective effects (*p* < 0.01), aligning with the previously noted improvement in cardiac functional parameters. There was no significant difference in the extent of improvement for these indices between the 7.5 mg/kg p-CT and 2.5 mg/kg Metoprolol groups (*p* > 0.05). Collectively, these data indicate that intravenous p-CT pretreatment effectively mitigates MIRI-induced cardiomyocyte damage and curtails the release of myocardial-specific proteins and intracellular enzymes into the bloodstream.

### 2.5. Pretreatment with p-CT Ameliorates Histological Damage in Myocardial Tissue of Rats with MIRI

**Figure 5:**
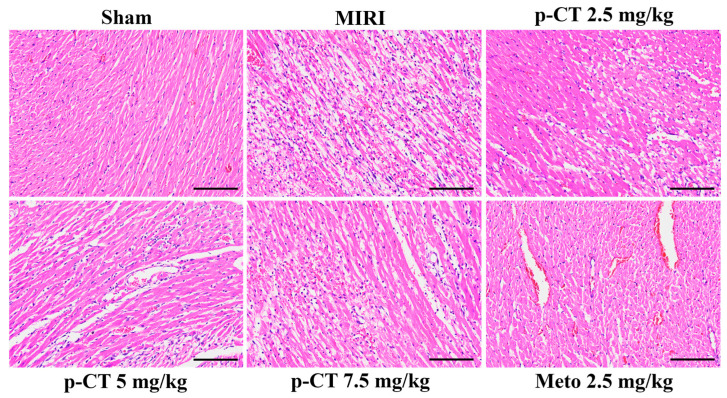
Histological evaluation of myocardial tissue across experimental rat groups (HE staining, ×200 magnification). Scale bar = 100 μm.

Histological assessment of myocardial tissue via hematoxylin and eosin (HE) staining is presented in Figure 5. In the Sham group, myocardial architecture was preserved, characterized by orderly, densely packed, and uniform cardiomyocyte fibers, regular nuclei, and an absence of interstitial edema or inflammatory cell infiltration. In contrast, the model group exhibited extensive disruption of myocardial structure, including the widespread disarray, fragmentation, and dissolution or necrosis of myocardial fibers; marked widening of intercellular spaces indicative of significant interstitial edema; and substantial infiltration of inflammatory cells, predominantly neutrophils. These findings confirm that MIRI induces severe histological damage. Pretreatment with p-CT significantly attenuated myocardial tissue injury in a dose-dependent manner compared to the model group. In the low-dose p-CT pretreatment group (2.5 mg/kg), myocardial fiber disorganization and interstitial edema were reduced relative to the model group, although localized inflammatory cell infiltration persisted. The medium-dose (5 mg/kg) and high-dose (7.5 mg/kg) p-CT pretreatment groups demonstrated the most pronounced improvement in myocardial pathology: cardiomyocyte fiber arrangement was more regular, fragmentation was minimized, and both interstitial edema and inflammatory cell infiltration were effectively suppressed, with tissue structure approaching that of the sham group. The positive control intervention with Metoprolol (2.5 mg/kg) also yielded significant cardioprotective effects, substantially reducing pathological damage. In summary, intravenous p-CT pretreatment mitigates MIRI-induced myocardial fiber disorder, cellular necrosis, interstitial edema, and inflammatory infiltration in a dose-dependent fashion.

### 2.6. Pretreatment with p-CT Markedly Diminishes Myocardial Infarct Size in Rats Subjected to Ischemia–Reperfusion Injury

**Figure 6:**
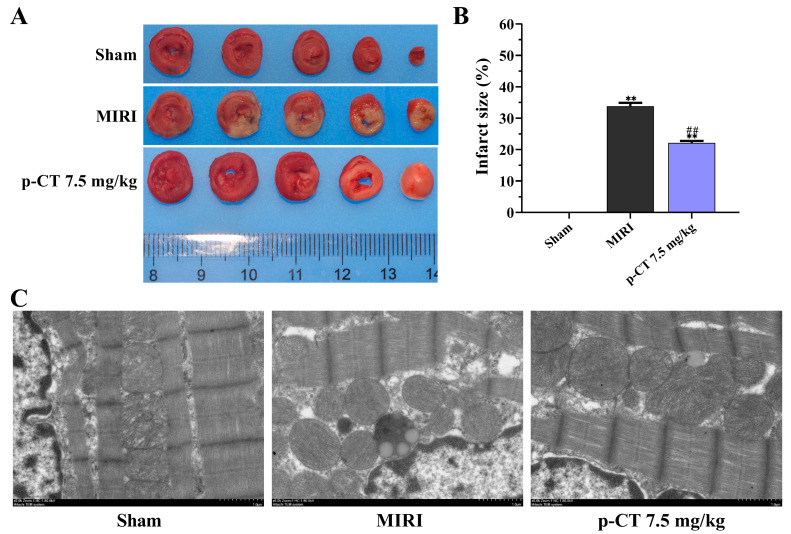
Impact of p-CT pretreatment on infarct size and ultrastructural alterations in rat cardiomyocytes. (**A**) Representative macroscopic photographs of TTC-stained myocardial tissues from each experimental group (red zones denote surviving myocardium; gray-white zones indicate infarcted regions). (**B**) Quantitative assessment of infarct size percentage (data presented as mean ± standard deviation, *n* = 3). One-way ANOVA followed by Tukey’s post hoc test: F(2, 6) = 398.1, *p* < 0.0001. ** *p* < 0.01 vs. sham group; ## *p* < 0.01 vs. MIRI group. (**C**) Representative transmission electron micrographs depicting ultrastructural modifications in cardiomyocytes across groups (scale bar = 1 μm).

To assess the cardioprotective properties of p-CT against MIRI, infarct size was determined through 2,3,5-triphenyltetrazolium chloride (TTC) staining, a standard technique for distinguishing viable from necrotic tissue. Figure 6A illustrates that myocardial sections from sham-operated rats exhibited a homogeneous deep red coloration without any pale infarct zones, indicating intact myocardium. Conversely, in the MIRI group, extensive gray-white regions were evident in the left ventricular anterior wall, reflecting substantial myocardial infarction. Intravenous pretreatment with p-CT (7.5 mg/kg) led to a notable amelioration of this damage; heart slices from the p-CT-pretreated group displayed conspicuously reduced gray-white infarct areas compared to the MIRI group. Quantitative evaluation of the infarct size percentage (Figure 6B) substantiated these morphological findings. Relative to the sham group, the MIRI group demonstrated a significant increase in infarct size percentage (*p* < 0.01). p-CT pretreatment at 7.5 mg/kg effectively suppressed this injury, resulting in a statistically significant reduction in infarct size percentage (*p* < 0.01). These outcomes demonstrate that p-CT pretreatment robustly curtails necrotic progression following myocardial ischemia–reperfusion and achieves a significant decrease in infarct dimensions.

### 2.7. Pretreatment with p-CT Markedly Ameliorates Ultrastructural Damage in Myocardial Cells Induced by Ischemia–Reperfusion Injury in Rats

To assess the morphological alterations of myocardial injury at the subcellular level, transmission electron microscopy was employed to examine the ultrastructure of rat myocardial cells across experimental groups (Figure 6C). In the sham group, myocardial cells displayed an intact cellular architecture, with myofibrils arranged in an orderly, compact pattern and clearly defined Z-lines. Mitochondria presented with normal morphology, uniform dimensions, distinct cristae, and a tightly packed arrangement, showing no signs of swelling or vacuolation. By contrast, myocardial tissue from the MIRI group exhibited pronounced disarray and fragmentation of myofibril organization, accompanied by a subset of mitochondria that were significantly swollen and enlarged. Within these affected mitochondria, cristae displayed fragmentation, dissolution, or complete loss, leading to the formation of characteristic vacuolar spaces. In the group pretreated with p-CT (7.5 mg/kg), the ultrastructural integrity of myocardial cells was substantially preserved. Relative to the MIRI group, myofibril alignment appeared more regular, with reduced rupture. Mitochondrial injury was notably mitigated: although mild swelling was observed in some organelles, the majority retained crisply defined cristae with a relatively organized architecture, and vacuolization was significantly diminished. Collectively, these observations indicate that pretreatment with p-CT effectively stabilizes mitochondrial membrane integrity and attenuates the disruption of myofibrillar structure in the setting of MIRI.

### 2.8. Label-Free Quantitative Proteomics Analysis Reveals That p-CT Preconditioning Reverses the Specific Protein Expression Profile Induced by MIRI

**Figure 7:**
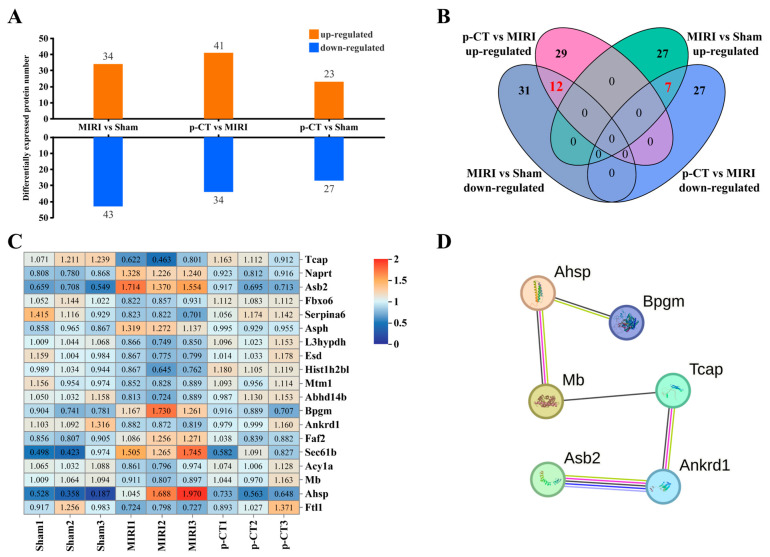
Label-free quantitative proteomics screening of p-CT reversing myocardial ischemia–reperfusion injury-related proteins. (**A**) Statistical summary of differentially expressed proteins among experimental groups. The bar chart displays the number of significantly upregulated and downregulated proteins between the MIRI group and the sham surgery group, the p-CT (7.5 mg/kg) group and the MIRI group, and the p-CT (7.5 mg/kg) group and the sham surgery group (screening threshold: fold change >1.2 or <0.83, *p* < 0.05). (**B**) Venn diagram analysis of differentially expressed proteins. This diagram presents the overlap of proteins among the three comparison groups, used to identify proteins specifically regulated by p-CT—those that were dysregulated in the MIRI model but restored to sham surgery group levels following p-CT intervention. (**C**) Heatmap display of proteins reversed by p-CT. The expression patterns and relative abundances of 19 proteins identified as targets of p-CT are shown in the sham surgery group, MIRI group, and p-CT pretreatment group. (**D**) Protein interaction network of reversed proteins. The interaction network analysis of the above 19 reversed proteins shows that 6 proteins form a significantly interacting network module.

To systematically investigate the molecular targets that may mediate the cardioprotective effects of p-CT, we performed label-free quantitative proteomics analysis on myocardial tissues from the sham, MIRI, and p-CT (7.5 mg/kg) preconditioning groups. All identified proteins and related peptide information are detailed in the Supplementary Material (Table S1). The number of differentially expressed proteins between groups is presented in Figure 7A: compared with the sham group, the MIRI group showed significant alterations in the expression of 77 proteins, of which 34 were upregulated and 43 were downregulated. In comparison with the MIRI group, the p-CT preconditioning group exhibited significant changes in 75 proteins, with 41 upregulated and 34 downregulated.

To precisely identify proteins specifically reversed by p-CT—i.e., proteins whose abnormal expression in MIRI was restored toward sham-like levels after p-CT intervention—a Venn diagram intersection analysis was conducted. As illustrated in Figure 7B, differentially expressed proteins from the “MIRI vs. Sham” comparison were intersected with those from the “p-CT vs. MIRI” comparison, while excluding proteins that remained significantly altered in the “p-CT vs. Sham” comparison. This approach ultimately identified 19 core p-CT-reversal proteins, which represent the most direct potential targets underlying p-CT’s protection. The expression patterns of these 19 reversal proteins are clearly displayed in the heatmap (Figure 7C): proteins that were up- or downregulated in the MIRI group tended to return to levels resembling the sham group in the p-CT preconditioning group, providing a visual confirmation of p-CT’s “reversal” effect. Notably, Tcap exhibited the most pronounced expression change among the differentially expressed proteins in “MIRI vs. Sham” and was strongly reversed by p-CT, suggesting it may be a key mediator of p-CT’s action.

To further clarify the functional relationships among these potential targets, a protein–protein interaction (PPI) network was constructed. As shown in Figure 7D, the analysis revealed that six of these proteins—Tcap, ankyrin repeat domain 1 (Ankrd1), Myoglobin (Mb), alpha-hemoglobin-stabilizing protein (Ahsp), bisphosphoglycerate mutase (Bpgm), and ankyrin repeat and socs box-containing protein 2 (Asb2)—form a tightly connected interaction sub-network, implying that they may cooperate in regulating specific biological pathways or cellular processes.

**Figure 8:**
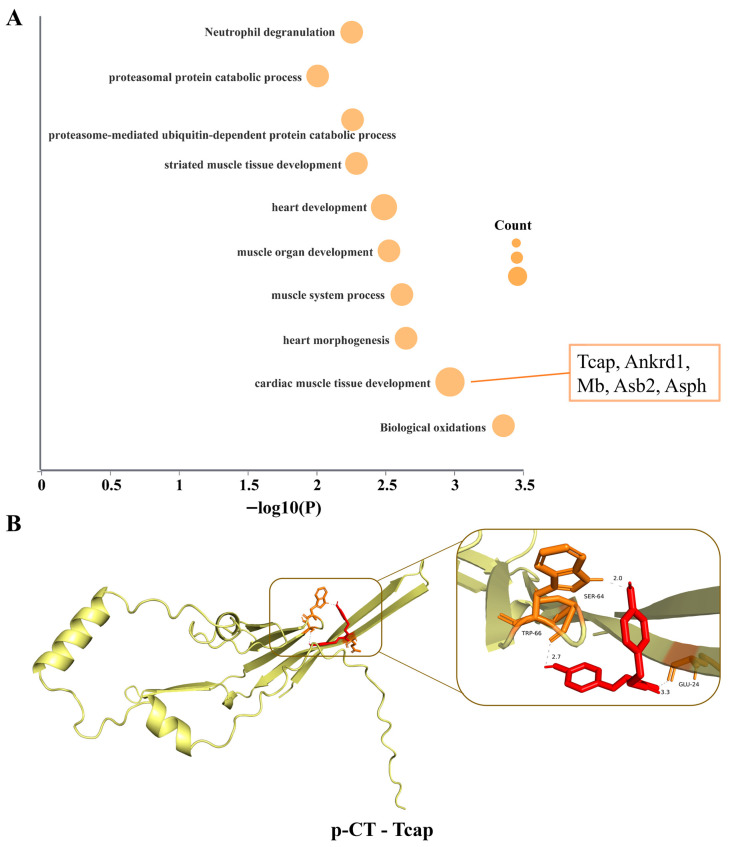
Gene Ontology (GO) enrichment analysis of p-CT reversing proteins and molecular docking studies with key targets. (**A**) Bubble chart of GO biological process enrichment analysis for 19 p-CT reversing proteins. The size of the bubbles represents the number of enriched proteins (Count), while the horizontal axis corresponds to the negative logarithm of the significance of enrichment (−log_10_(*p*) value). (**B**) Molecular docking simulation results of p-CT with the candidate target protein Tcap. This shows the three-dimensional binding conformation of the p-CT molecule (represented by a stick model) with the target protein (represented by a surface or ribbon model). The figure labels the key interacting amino acid residues and the predicted binding free energy (ΔG, in kcal/mol).

To explore the potential biological functions of the 19 p-CT-reversal proteins, we performed Gene Ontology (GO) enrichment analysis. The results, presented in Figure 8A, demonstrate that these proteins are significantly enriched in multiple biological processes associated with the pathophysiology and repair of myocardial ischemia–reperfusion injury. The most significantly enriched categories included “biological oxidations,” “cardiac muscle tissue development,” and “heart morphogenesis.” Among these, “cardiac muscle tissue development” contained the largest number of proteins, including Tcap, Ankrd1, Mb, Ahsp, and Asb2. To further examine the potential therapeutic targets of p-CT within this protein set, molecular docking analysis was carried out, focusing on two proteins closely linked to myocardial structure and function that also appear in the interaction network—Tcap and Ankrd1—with particular attention to Tcap given its notable reversal effect. As shown in Figure 8B, molecular docking predicted that p-CT can bind to a specific pocket in Tcap with a binding free energy of –6.3 kcal/mol, potentially forming interactions with amino acid residues Glu-24, Ser-64, and Trp-66. In contrast, docking with Ankrd1 yielded a binding energy of –5.6 kcal/mol, but the model did not reveal a stable interaction site involving specific amino acids.

In summary, through proteomic analysis, this study identified 19 key proteins whose aberrant expression induced by MIRI was specifically reversed by p-CT. These proteins were significantly enriched in processes such as cardiac development and biological oxidation. Molecular docking further suggested that p-CT exhibits high binding affinity with the candidate protein Tcap, indicating that Tcap could serve as a potential molecular target contributing to the cardioprotective effects of p-CT.

### 2.9. Pretreatment with p-CT Reverses the Downregulation of Tcap and Ankrd1 Protein Expression Induced by MIRI in Rat Cardiac Tissue

**Figure 9:**
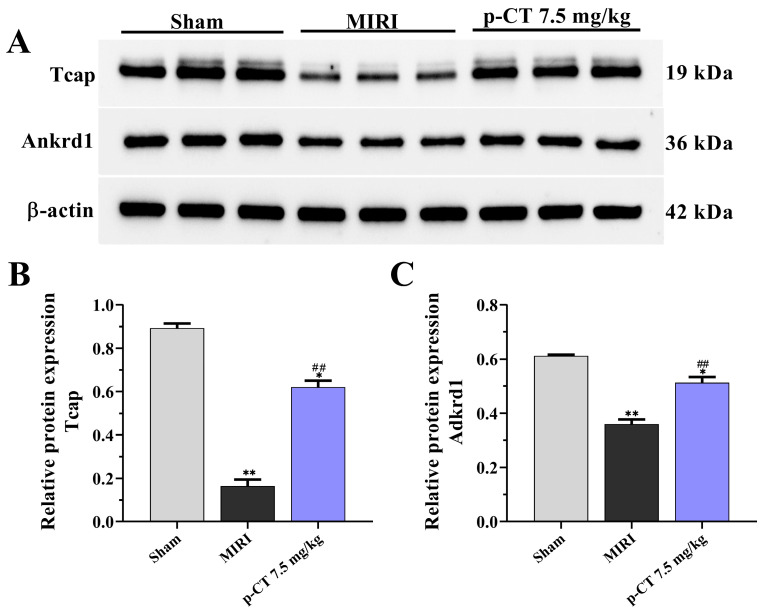
Effect of p-CT pretreatment on protein expression levels of Tcap and Ankrd1 in rat myocardial tissue. (**A**) Representative Western blot bands. (**B**) Semi-quantitative statistical analysis of Tcap protein expression levels. Data are presented as mean ± SD (*n* = 3). One-way ANOVA followed by Tukey’s post hoc test: F(2, 6) = 185.8, *p* < 0.0001. * *p* < 0.05, ** *p* < 0.01 vs. sham group; ## *p* < 0.01 vs. MIRI group. (**C**) Semi-quantitative statistical analysis of Ankrd1 protein expression levels. Data are presented as mean ± SD (*n* = 3). One-way ANOVA followed by Tukey’s post hoc test: F(2, 6) = 52.4, *p* < 0.001. * *p* < 0.05, ** *p* < 0.01 vs. sham group; ## *p* < 0.01 vs. MIRI group.

To validate the proteomic screening results at the protein level and further investigate changes in the expression of the potential target proteins Tcap and Ankrd1 in myocardial tissue, this study employed Western blot analysis to measure the expression levels of these proteins in rat cardiac samples. As illustrated in Figure 9, the results demonstrate that, compared to the sham group, the protein expression levels of both Tcap and Ankrd1 were significantly downregulated in myocardial tissue from MIRI rats (*p* < 0.01). This finding aligns with the conclusions derived from label-free quantitative proteomics, confirming that myocardial ischemia–reperfusion injury disrupts the expression balance of structural proteins in the heart. Intravenous pretreatment with p-CT (7.5 mg/kg) effectively inhibited the MIRI-induced downregulation of Tcap and Ankrd1 protein expression. Figure 9A–C show that, relative to the MIRI group, the protein expression levels of Tcap and Ankrd1 in the p-CT pretreatment group were significantly increased (*p* = 0.0002, *p* = 0.0051). Although the expression levels of both proteins in the p-CT pretreatment group remained lower than those in the sham group (*p* < 0.05) and did not completely normalize, they still exhibited a significant increase when compared to the MIRI group (*p* < 0.01). These data indicate that p-CT pretreatment can partially restore the expression of these two key proteins following myocardial ischemia–reperfusion injury.

## 3. Discussion

This study systematically examined the protective effects and underlying mechanisms of the natural compound p-CT against MIRI by integrating in vitro cellular models, in vivo animal experiments, and label-free quantitative proteomics. The key findings demonstrate that p-CT exerts potent cardioprotective effects across multiple dimensions, with its mechanism of action likely closely associated with the regulation of Tcap, a critical sarcomeric protein.

Our research is the first to establish that p-CT provides significant myocardial protection in both in vitro hypoxia/reoxygenation injury models and in vivo rat models of myocardial ischemia–reperfusion injury. This protective activity is evident not only in enhanced cell viability and improved cardiac function parameters, but also in the amelioration of histopathological damage, reduction in myocardial infarct size, and preservation of cellular ultrastructural integrity. Importantly, through label-free quantitative proteomics coupled with bioinformatics analyses, we identified the Titin-cap protein Tcap as a key molecular target of p-CT. The upregulation of Tcap expression represents a pivotal mechanism through which p-CT likely mediates its cardioprotective function.

Our investigation offers comprehensive experimental validation across multiple levels, demonstrating the cardioprotective properties of p-CT. First, in vitro assessments revealed that p-CT, at a non-toxic concentration range of 5–10 μM, dose-dependently improved the viability of neonatal rat cardiomyocytes subjected to hypoxia/reoxygenation injury. Concurrently, p-CT markedly suppressed the release of established injury biomarkers LDH and CK-MB. Since elevated serum levels of LDH and CK-MB correlate positively with the extent of myocardial damage [27], these data provide direct evidence of p-CT’s ability to protect cardiomyocytes from injury. Second, whole-animal studies comprehensively corroborated the efficacy of p-CT. Cardiac functional analyses showed that pretreatment with p-CT significantly ameliorated ventricular systolic and diastolic dysfunction induced by myocardial ischemia–reperfusion injury (MIRI), as reflected by increased left ventricular systolic pressure (LVSP) and maximum rates of pressure development and decline (±dp/dtmax), along with decreased left ventricular end-diastolic pressure (LVEDP). Biochemically, serum levels of the cardiac-specific injury markers cardiac troponin T (cTnT) and myoglobin (Mb), as well as LDH and CK-MB, were substantially reduced, confirming attenuation of myocardial injury. Troponin is regarded as the preferred diagnostic biomarker for myocardial injury owing to its high cardiac specificity and sensitivity, especially in acute myocardial infarction. Myoglobin, a small oxygen-binding protein abundant in cardiac muscle, is rapidly released upon cardiomyocyte damage and serves as an early indicator [28]. The combined measurement of cTnT, Mb, LDH, and CK-MB thus allows a comprehensive assessment to be made of the timing and severity of myocardial injury. Histopathological examination further demonstrated that p-CT effectively mitigated disordered myocardial fiber arrangement, interstitial edema, and inflammatory cell infiltration. Triphenyltetrazolium chloride (TTC) staining confirmed that p-CT significantly reduced infarct size. Together, these coherent functional, biochemical, and morphological findings form a complete chain of evidence validating the in vivo cardioprotective role of p-CT. Finally, transmission electron microscopy elucidated the ultrastructural basis of p-CT’s protection at the subcellular level. p-CT pretreatment effectively attenuated MIRI-induced mitochondrial swelling, the disruption of cristae architecture, and the disorganization of myofibril arrangement. As the primary cellular energy producers and key regulators of apoptosis, the maintenance of mitochondrial structural integrity is critical for cardiomyocyte survival [29]. Hence, the preservation of the mitochondrial ultrastructure likely represents an important mechanism through which p-CT improves cardiomyocyte energy metabolism, inhibits cell death, and ultimately confers cardioprotection.

To further investigate the underlying mechanisms of p-CT, we performed label-free quantitative proteomic profiling. The comparative analysis of protein expression patterns across the sham, MIRI, and p-CT treatment groups revealed 19 differentially expressed proteins that were specifically reversed following p-CT administration. Gene Ontology (GO) enrichment analysis indicated that these proteins were significantly associated with biological processes such as “heart development,” “myocardial tissue development,” and “biological oxidation.” These findings imply that the cardioprotective action of p-CT is not mediated through a single molecular target, but likely involves the modulation of a protein network linked to cardiac structural integrity, developmental pathways, and energy metabolism, thereby producing a synergistic therapeutic effect. Among the 19 identified proteins, four have established relevance to myocardial ischemia–reperfusion injury or cardiac damage. Ferritin light chain (Ftl1) serves as a crucial intracellular iron-storage protein. During myocardial ischemia, toxin exposure, or cellular stress, the downregulation or impaired function of Ftl1 reduces iron-buffering capacity, leading to free iron overload [30]. This excess iron promotes lipid peroxidation and drives ferroptosis, ultimately exacerbating myocardial injury [31]. Myoglobin is a well-recognized early and sensitive biomarker of acute myocardial injury, including ischemia–reperfusion events [32,33]. Ankyrin Repeat Domain 1 (Ankrd1) is a multifunctional protein expressed in cardiac and skeletal muscle, localized to both the nucleus and sarcomere. In models of hypoxia-induced apoptosis in rat embryonic cardiomyocytes, the induction of the pro-apoptotic gene GADD153 results in the transcriptional downregulation of Ankrd1 [34]. Tcap (Titin-cap) is a Z-disc protein predominantly expressed in striated muscle (cardiac and skeletal). It plays a central role in maintaining sarcomere architecture, mechanical signaling, ion channel regulation, and cellular metabolic and stress responses. Substantial evidence links mutations in the Tcap gene to various cardiomyopathies, including hypertrophic cardiomyopathy, dilated cardiomyopathy, and restrictive phenotype hypertrophic cardiomyopathy [35,36,37,38,39].

Within this protein interaction network, our investigation centers on the Tcap protein for the following reasons. First, regarding expression dynamics, Tcap is significantly downregulated in the MIRI group, whereas its expression is most robustly restored following p-CT intervention. This pattern identifies Tcap as a molecule highly sensitive to both injury and therapeutic treatment. Second, from a functional perspective, Tcap (Titin-cap protein) is a core structural component of the Z-disc in muscle sarcomeres, essential for maintaining the mechanical integrity and stability of cardiomyocyte sarcomeres and for transmitting mechanical stress signals. Prior studies have established that mutations or the aberrant expression of the Tcap gene are closely associated with diseases such as dilated cardiomyopathy and heart failure [35,36]. In the present study, Western blot analysis confirmed that MIRI induces the downregulation of Tcap protein expression in myocardial tissue, and that p-CT effectively reverses this change. This result aligns logically with the observed improvements in cardiac function—which depends on an intact contractile apparatus—and with the protection of myofibril ultrastructure, suggesting that p-CT may upregulate Tcap to stabilize the contractile framework of cardiomyocytes and thereby counteract structural damage induced by ischemia–reperfusion. Third, molecular docking simulations provide theoretical support for a direct interaction between p-CT and Tcap. Computational modeling indicates that p-CT can bind with high affinity within specific pockets of the Tcap protein, engaging amino acid residues Glu-24, Ser-64, and Trp-66, which implies a plausible direct binding event. Notably, another protein in the network, Ankrd1, though also influenced in expression by p-CT, did not exhibit stable or specific binding sites with p-CT in docking simulations. This suggests that Ankrd1 upregulation may not be a direct consequence of p-CT binding, but rather that Ankrd1 is indirectly modulated as an interaction partner or downstream effector of Tcap. Its precise role warrants further exploration.

Notably, in our in vivo investigations, the administration of p-CT at medium to high doses (5–7.5 mg/kg) yielded cardioprotective effects that were comparable to those of the classical β1-adrenergic receptor antagonist metoprolol (2.5 mg/kg). This was reflected in analogous enhancements in cardiac functional indices and comparable attenuation of serum markers of myocardial injury. Although both compounds mitigated MIRI, it is critical to underscore that p-CT, a naturally occurring amide alkaloid, is anticipated to act via a divergent mechanistic pathway. In contrast to metoprolol, which chiefly exerts its effects through β1-adrenoceptor blockade to diminish cardiac workload and oxygen consumption, our integrated proteomic and molecular docking analyses indicate that p-CT may function, at least in part, via the upregulation of the sarcomeric Z-disc protein Tcap. This mechanistic distinction suggests that p-CT could embody a novel structure-centric cardioprotective strategy—one that targets the preservation of myocardial architectural integrity. Consequently, it may offer a complementary or alternative paradigm to conventional neurohormonal modulation for the therapeutic management of ischemia–reperfusion injury.

This study has several limitations that should be addressed in future research. First, target validation requires further refinement. While our proteomic profiling and Western blot validation consistently demonstrate that the cardioprotective benefits of p-CT are linked to the restoration of Tcap levels following MIRI-induced downregulation, and molecular docking simulations indicate a plausible direct binding interaction, these findings alone do not confirm a causal relationship. Subsequent studies should directly confirm their physical interaction using biophysical techniques such as surface plasmon resonance and isothermal titration calorimetry [40]. Moreover, functional rescue experiments—through the overexpression or knockdown of Tcap in cardiomyocyte models—are necessary to clarify whether Tcap is indispensable within the p-CT-mediated cardioprotective signaling pathway. Second, downstream signaling pathways have not been fully elucidated. The effects of p-CT-induced Tcap upregulation on sarcomere assembly, mechanical signal transduction (e.g., interactions with MAPK and PI3K/Akt pathways), or mitochondrial function-specific downstream networks require comprehensive molecular and cellular biology experiments for deeper exploration. Third, the selection of p-CT doses for the in vivo studies (2.5, 5, and 7.5 mg/kg) was derived from preliminary range-finding experiments and the extrapolation of effective concentrations observed in cellular models. A notable limitation is the lack of pharmacokinetic data, as we did not assess plasma concentrations of p-CT following intravenous administration. Consequently, the correlation between the administered dose, the achieved systemic exposure, and the observed cardioprotective effects remains unverified. Future investigations should incorporate pharmacokinetic profiling to define the optimal therapeutic window and determine the bioavailability of p-CT. Additionally, the potential contributions and synergistic effects of other proteins modulated by p-CT in its overall mechanism of action warrant further investigation.

In summary, this study indicates that p-CT is a promising candidate drug for the treatment of myocardial ischemia–reperfusion injury. Its cardioprotective effects are reflected in significant improvements in cell viability, cardiac function, tissue pathology, and ultrastructure. Mechanistically, p-CT likely exerts cardioprotection by upregulating the expression of the key sarcomeric protein Tcap, thereby stabilizing the structural integrity of cardiomyocytes. This provides an important molecular explanation for its protective role.

## 4. Materials and Methods

### 4.1. Experimental Animals

Healthy adult male Sprague Dawley rats (7–8 weeks old), with body weights of 250 ± 20 g, were obtained from the Experimental Animal Center of Guizhou University of Traditional Chinese Medicine. The animal use protocol was approved under license number SYXK (Qian) 2021-0005. All animals were housed in the standard animal facility of the Basic Medical Experimental Teaching Demonstration Center at Guizhou University of Traditional Chinese Medicine. Environmental conditions were maintained at a temperature of 22 ± 2 °C, relative humidity of 50 ± 10%, and a 12 h light/dark cycle. The rats had ad libitum access to standard rodent chow and drinking water. After one week of acclimatization, the rats were randomly assigned into groups of 10 each, with 5 rats per cage. They then received intravenous administration of the designated compounds and underwent surgery to establish the myocardial ischemia–reperfusion injury model.

### 4.2. Reagents

N-*p*-*trans*-Coumaroyltyramine (HY-N2230), Metoprolol (HY-17503), and N-Acetyl-L-cysteine (HY-B0215) were purchased from MedChemExpress Co., Ltd. (Monmouth Junction, NJ, USA). The Cell Counting Kit (CCK-8 Kit) (C0038), RIPA lysis buffer (P0013K), protease inhibitor cocktail (P1048), and BCA Protein Assay Kit (P0010) were purchased from Beyotime Biotechnology Co., Ltd. (Shanghai, China). Lactate dehydrogenase (LDH) assay kit (A020-2-2), Creatine kinase-MB (CK-MB) assay kit (H197-1-2), rat cardiac troponin T (cTnT) ELISA kit (H149-4-2), and rat myoglobin (Mb) ELISA kit (H150-1-1) were all obtained from Nanjing Jiancheng Bioengineering Institute (Nanjing, Jiangsu, China). TTC (2,3,5-Triphenyltetrazolium chloride) staining solution (G3004) was purchased from Beijing Solarbio Science & Technology Co., Ltd. (Beijing, China). Tcap monoclonal antibody (19 kDa, A22731) was obtained from ABclonal Biotechnology Co., Ltd. (Wuhan, Hubei, China). Ankrd1 polyclonal antibody (36 kDa, 11427-1-AP) was purchased from Proteintech Group, Inc. (Wuhan, Hubei, China). Rat cardiomyocyte complete culture medium (CM-R073) was obtained from Wuhan Procell Life Technology Co., Ltd. (Wuhan, Hubei, China).

### 4.3. Isolation and Culture of Neonatal Rat Cardiomyocytes, and Establishment and Treatment of the Hypoxia/Reoxygenation Injury Model

Primary Sprague Dawley (SD) neonatal rat cardiomyocytes (catalog number: CP-R073) were obtained from Wuhan Procell Life Technology Co., Ltd. (Wuhan, Hubei, China). Cells were plated at a density of 5 × 10^5^ cells/mL in complete rat cardiomyocyte culture medium and maintained under standard culture conditions in a humidified incubator at 37 °C with 5% CO_2_. To determine the non-toxic working concentration range for p-CT, the cells were treated with 0.1, 1, 10, 50, 100, or 200 μM p-CT (*n* = 8 per group) for 24 h, after which cell viability was evaluated using the CCK-8 assay.

For the hypoxia/reoxygenation (H/R) model, the existing culture medium was removed and replaced with a hypoxic medium that had been pre-equilibrated with a gas mixture of 94% N_2_, 1% O_2_, and 5% CO_2_. The cells were then transferred to a tri-gas incubator set to the same gas composition (94% N_2_, 1% O_2_, 5% CO_2_) and incubated at 37 °C for 6 h to induce hypoxic injury. Following the hypoxic period, fresh complete culture medium was added, and the cells were returned to normoxic conditions (37 °C, 5% CO_2_) for 12 h of reoxygenation [41].

Prior to H/R induction, the cells were pretreated for 2 h with varying concentrations of p-CT (0.1, 1, 5, 10, 20, or 50 μM) (*n* = 8 per group) or with the positive control N-acetylcysteine (NAC, 200 μM) (*n* = 8). After completion of the H/R protocol, cell viability was again assessed using the CCK-8 method. To evaluate the effects of the drug on myocardial cell injury, the cells were pretreated with different concentrations of p-CT (6, 8, 10 μM) (*n* = 8 per group) for 2 h. The culture supernatant was collected for the measurement of lactate dehydrogenase (LDH) and creatine kinase-MB (CK-MB) activity.

### 4.4. Establishment and Grouping of the Rat Myocardial Ischemia–Reperfusion Injury Model

The experimental rats were anesthetized by the intraperitoneal injection of sodium pentobarbital (45 mg/kg). Once anesthesia was effective, tracheal intubation was performed and connected to a small-animal ventilator to maintain spontaneous respiration. A skin incision was made on the left side of the sternum, followed by the layer-by-layer blunt dissection of subcutaneous tissue and muscle. A thoracotomy was carried out through the 4th intercostal space to fully expose the heart. Approximately 2 mm below the inferior margin of the left atrial appendage, a 5-0 surgical suture was passed underneath the left anterior descending coronary artery. Both ends of the suture were threaded through a segment of polyethylene tubing to create a reversible snare. Tightening the suture together with the polyethylene tube occluded coronary blood flow, inducing regional myocardial ischemia. After 30 min of ischemia, the ligature was released to restore arterial patency, allowing coronary reperfusion for 2 h, thereby establishing the myocardial ischemia–reperfusion injury (MIRI) model [42].

All rats were randomly assigned to six groups, with 10 rats per group:

(1) Sham-operated group: Only the threading procedure was performed without coronary artery ligation.
(2) MIRI model group: The MIRI model was established, and a solvent vehicle was administered via tail-vein injection 30 min before ischemia.
(3) Low-dose p-CT pretreatment group: 2.5 mg/kg p-CT solution was injected via the tail vein 30 min before ischemia.
(4) Medium-dose p-CT pretreatment group: 5.0 mg/kg p-CT solution was injected via the tail vein 30 min before ischemia.
(5) High-dose p-CT pretreatment group: 7.5 mg/kg p-CT solution was injected via the tail vein 30 min before ischemia.
(6) Positive drug control group: Metoprolol (2.5 mg/kg) was administered via tail-vein injection 30 min before ischemia.

In the establishment of the sham or MIRI model, one rat from each group was excluded due to massive hemorrhage from the ligation of the left anterior descending coronary artery or early onset of ventricular fibrillation during reperfusion, resulting in a final effective sample size of *n* = 9 for each group. The assessment of cardiac function and serum biological markers ultimately had a sample size of *n* = 9 per group; morphological and ultrastructural observations of myocardial tissue had *n* = 3 per group; myocardial infarction area measurements had *n* = 3 per group; proteomics analysis had *n* = 3 per group; and Western blot analysis had *n* = 3 per group. The same protein samples were used for both proteomics analysis and Western blot analysis.

### 4.5. Assessment of Cardiac Function and Serological Biomarkers

Following anesthesia, the right carotid artery of each rat was cannulated. The arterial catheter was linked to a BL-420s biological signal acquisition system (Chengdu TME Technology Co., Ltd., Chengdu, China) to monitor key hemodynamic parameters. These included left ventricular systolic pressure (LVSP), left ventricular end-diastolic pressure (LVEDP), and the maximum rates of ventricular pressure rise and decline (±dp/dtmax).

Upon completion of the reperfusion phase, whole-blood samples were obtained via the abdominal aorta. After clotting at room temperature, the samples were centrifuged at 2500 rpm for 10 min at 4 °C to isolate serum. According to the respective assay kit protocols, serum levels of lactate dehydrogenase (LDH) and creatine kinase-MB (CK-MB) activity were determined, along with the concentrations of cardiac troponin T (cTnT) and myoglobin (Mb).

### 4.6. Morphology and Ultrastructural Observation of Myocardial Tissue

Myocardial tissue samples were harvested from the risk region of the left ventricular infarction site. A portion of these samples was promptly fixed in 4% paraformaldehyde solution and maintained at 4 °C for 24 h to ensure optimal morphological preservation. Following fixation, the tissues underwent sequential dehydration, clearing, and paraffin embedding. Sections were cut to a thickness of 5 μm using a microtome, stained with hematoxylin and eosin (HE), and examined under an optical microscope (BX53, Olympus, Tokyo, Japan) to assess myocardial morphology, with digital images captured for documentation.

For ultrastructural evaluation, another subset of myocardial tissue was utilized. Approximately 1 mm^3^ fragments were rapidly immersed in pre-chilled 2.5% glutaraldehyde fixative and fixed overnight at 4 °C. The samples were then post-fixed with 1% osmium tetroxide, dehydrated through a graded ethanol series, and embedded in epoxy resin. Ultrathin sections (70 nm thick) were prepared, double-stained with uranyl acetate and lead citrate, and ultimately visualized using a transmission electron microscope (Hitachi H-600, Tokyo, Japan) to examine the fine structural details of cardiomyocytes.

### 4.7. Measurement of Myocardial Infarction Area

Following reperfusion, the heart was promptly excised and rinsed with ice-cold physiological saline to eliminate residual blood. Subsequently, the heart was placed at –20 °C for a brief freeze (10 min) to firm the tissue for easier sectioning. The heart was sliced along the transverse (short-axis) plane into uniform sections of about 2 mm thickness. The tissue slices were completely immersed in 1% triphenyltetrazolium chloride (TTC) staining solution and incubated at 37 °C in a light-protected environment for 15 min. During incubation, viable myocardium, which retains active dehydrogenases, stains a characteristic brick red, whereas infarcted myocardium, devoid of enzyme activity, remains pale gray-white. After staining, the slices were photographed under standardized conditions using a digital camera for documentation. Excess surface moisture was gently blotted from the slices with filter paper. Under a stereomicroscope, the infarcted (gray-white) zones were meticulously separated from the non-infarcted (brick red) regions using fine dissecting tools. The total myocardial slice and the isolated infarcted tissue were weighed separately. The myocardial infarction area was then expressed as a percentage, calculated using the formula (Infarcted tissue weight/Total myocardial weight × 100%) [43].

### 4.8. Label-Free Quantitative Proteomics Analysis

In this study, myocardial tissue samples from rats in the sham group, MIRI group, and p-CT (7.5 mg/kg) pretreatment group were collected (*n* = 3 per group). The samples were submitted to Hangzhou Jingjie Biological Technology Co., Ltd. for label-free quantitative proteomic profiling. The experimental workflow included tissue lysis, total protein extraction, trypsin digestion, and subsequent analysis of the resulting peptide mixtures via liquid chromatography–tandem mass spectrometry (LC-MS/MS). Raw MS data were processed with MaxQuant software (version v1.6.6.0), and proteins were identified by searching against the UniProt Rattus norvegicus_10116_PR database. Differentially expressed proteins were defined based on the following thresholds: fold change >1.2 for upregulation or <0.83 for downregulation, with a *p*-value < 0.05. Venn diagram analysis of the differential proteins was then performed using the CNSknowall online platform (https://cnsknowall.com/#/HomePage?MTAw=NDQyNzk= (accessed on 21 April 2025)), a comprehensive tool for biomedical data analysis and visualization. Proteins that exhibited opposite expression trends in the “MIRI vs. Sham” and “p-CT vs. MIRI” comparisons, while showing no significant change in “p-CT vs. Sham,” were designated as “p-CT reverse proteins.” This subset reflects the capacity of p-CT pretreatment to reverse MIRI-induced alterations in protein expression.

### 4.9. Bioinformatics Analysis and Molecular Docking

For the 19 selected p-CT reversal proteins, a protein–protein interaction (PPI) network was constructed utilizing the STRING 12.0 database (https://string-db.org/). Gene Ontology (GO) functional enrichment analysis was performed using the Metascape online analysis platform (http://metascape.org), with a significance threshold set at *p* < 0.05. The results of the enrichment analysis were visualized as a bubble chart using the CNSknowall online tool. The analysis result of the clustering heatmap plot was generated using the R software (version 4.3.1) packages “dendextend” via CNSknowall. The three-dimensional structures of Tcap (Uniprot ID: A6HIS0) and Ankrd1 (Uniprot ID: Q8R560) were downloaded from the Uniprot database (https://www.uniprot.org/). Molecular docking simulations were conducted using the CB-Dock2 online tool (https://cadd.labshare.cn/cb-dock2/php/index.php (accessed on 23 April 2025)). The 3D structure of p-CT was constructed using ChemDraw v23.1.1.3 software and underwent energy minimization. The binding mode was visualized using PyMOL 2.3.0 software.

### 4.10. Western Blot Analysis

Myocardial tissue samples were homogenized on ice using a tissue grinder with pre-cooled RIPA lysis buffer supplemented with a protease inhibitor cocktail at a tissue-to-buffer ratio of 1:10 (*w*/*v*). The homogenate was centrifuged at 12,000× *g* for 15 min at 4 °C, after which the supernatant was carefully collected. Total protein concentration in the supernatant was determined using a BCA protein assay kit. For each sample, 40 μg of total protein was mixed with loading buffer, denatured by boiling, and separated by SDS-PAGE on a 10% polyacrylamide resolving gel. Following electrophoresis, proteins were transferred onto a PVDF membrane. The membrane was blocked with 5% skim milk prepared in TBST for 1 h at room temperature. After blocking, the membrane was incubated overnight at 4 °C with gentle shaking in primary antibodies diluted in antibody diluent: anti-Tcap (1:1000), anti-Ankrd1 (1:1000), and anti-β-actin (1:3000) as an internal control. The next day, the membrane was washed three times with TBST, 10 min per wash, followed by incubation with an HRP-conjugated secondary antibody corresponding to the host species of the primary antibody (dilution 1:5000) for 1 h at room temperature with shaking. After secondary antibody incubation, the membrane was thoroughly washed again with TBST. Protein bands were visualized using an ECL chemiluminescent substrate and imaged with a Tanon-5200 chemiluminescence imaging system (Shanghai, China). Semi-quantitative analysis was performed using ImageJ software (Version 1.53) by measuring the grayscale values of target protein bands and the corresponding β-actin bands. Relative protein expression levels were calculated as the ratio of the target protein band intensity to that of β-actin.

### 4.11. Statistical Analysis

Continuous variables are expressed as mean ± standard deviation (mean ± SD). GraphPad Prism version 9.0 (GraphPad Software, Boston, MA, USA) was utilized for all statistical computations. Comparisons between two independent groups were performed using Student’s *t*-test. For comparisons involving more than two groups, one-way analysis of variance (ANOVA) was applied. When the assumption of homogeneity of variances was satisfied, Tukey’s post hoc test was used for multiple comparisons following a significant ANOVA result. A two-tailed *p*-value of less than 0.05 was defined as the threshold for statistical significance.

## 5. Conclusions

This study establishes, for the first time, the significant cardioprotective efficacy of the natural compound N-*p*-*trans*-Coumaroyltyramine (p-CT) against MIRI, employing an integrated multi-level approach encompassing cellular assays, animal models, and omics technologies. At the cellular level, p-CT demonstrated a dose-dependent capacity to mitigate hypoxia/reoxygenation-induced cardiomyocyte injury. In animal models, pretreatment with p-CT markedly improved cardiac functional parameters in MIRI rats, reduced circulating biomarkers of myocardial damage, attenuated histopathological injury and ultrastructural disruption in cardiac tissue, and significantly limited myocardial infarct size. Mechanistically, label-free quantitative proteomics coupled with bioinformatics analysis identified that p-CT specifically normalizes the dysregulated expression of 19 key proteins induced by MIRI, which are predominantly enriched in biological pathways related to cardiac development. Among these, Tcap emerged as a pivotal candidate target, exhibiting significant downregulation following MIRI—an effect that was effectively reversed by p-CT treatment. Molecular docking studies further predicted a stable and favorable binding interaction between p-CT and the Tcap protein. Subsequent Western blot analysis confirmed that p-CT upregulates Tcap expression at the protein level. In conclusion, this research identifies p-CT as a promising cardioprotective agent, whose mechanism likely involves targeting the Tcap protein and restoring its expression to preserve cardiomyocyte structural and functional integrity. These findings provide a substantial experimental basis for the further development of p-CT as a lead compound in the therapeutic strategy against myocardial ischemia–reperfusion injury.

## Data Availability

The original contributions presented in this study are included in the article/Supplementary Materials. Further inquiries can be directed to the corresponding author.

## Supplementary Materials

The following supporting information can be downloaded at https://www.mdpi.com/article/10.3390/ijms27083523/s1.

## Abbreviations

The following abbreviations are used in this manuscript:

p-CT	N-*p*-*trans*-Coumaroyltyramine
MIRI	Myocardial ischemia–reperfusion injury
MLKL	Mixed lineage kinase domain-like pseudokinase
H/R	Hypoxia/reoxygenation
LDH	Lactate dehydrogenase
CK-MB	Creatine kinase isoenzyme MB
cTnT	Cardiac troponin T
Mb	Myoglobin
LVSP	Left ventricular systolic pressure
LVEDP	Left ventricular end-diastolic pressure
+dp/dtmax	Maximum rate of left ventricular pressure increase
−dp/dtmax	Maximum rate of left ventricular pressure decrease
HE	Hematoxylin and eosin
TTC	2,3,5-triphenyltetrazolium chloride
Tcap	Titin-cap
Ankrd1	Ankyrin Repeat Domain 1
Ahsp	Alpha-hemoglobin-stabilizing protein
Bpgm	Bisphosphoglycerate mutase
Asb2	Ankyrin repeat and SOCS box-containing protein 2
AMI	Myocardial infarction
AKT1	AKT Serine/Threonine Kinase 1
EGFR	Epidermal growth factor receptor
CCND1	Cyclin D1
PI3K	Phosphatidylinositol 3-Kinase
MAPK	Mitogen-Activated Protein Kinase
GO	Gene Ontology
